# Development of Eosinophilic Fasciitis during Infliximab Therapy for Psoriatic Arthritis

**DOI:** 10.1155/2016/7906013

**Published:** 2016-05-11

**Authors:** Richard Hariman, Payal Patel, Jennifer Strouse, Michael P. Collins, Ann Rosenthal

**Affiliations:** ^1^Division of Rheumatology, Department of Medicine, Medical College of Wisconsin, 9200 W. Wisconsin Avenue, Milwaukee, WI 53226, USA; ^2^Department of Neurology, Medical College of Wisconsin, 9200 W. Wisconsin Avenue, Milwaukee, WI 53226, USA

## Abstract

Eosinophilic fasciitis (EF) is a rare disorder involving chronic inflammation of the fascia and connective tissue surrounding muscles, nerves, and blood vessels. While its pathogenesis is not entirely understood, this disorder is thought to be autoimmune or allergic in nature. We present here a case of a 59-year-old male who developed peripheral eosinophilia and subsequent eosinophilic fasciitis during treatment with infliximab. To our knowledge, eosinophilic fasciitis has not been previously described in patients during treatment with an inhibitor of tumor necrosis factor *α*.

## 1. Introduction

Eosinophilic fasciitis is a rare disorder involving chronic inflammation of fascia and connective tissue surrounding muscles, nerves, and blood vessels [1]. We report a case of eosinophilic fasciitis in a 59-year-old male patient undergoing treatment with infliximab for psoriatic arthritis. Infliximab infusion triggered eosinophilic fasciitis three months after his medication dose was increased from 3 mg/kg to 5 mg/kg. To our knowledge, eosinophilic fasciitis has not been described in patients during therapy with a tumor necrosis factor-*α* (TNF-*α*) inhibitor.

## 2. Case Description

Mr. E was a 59-year-old former mechanic and current smoker with psoriatic arthritis who presented to the hospital with a 2-month history of worsening lower extremity swelling, arthralgias of his hands and feet, and new onset of muscle pain. He had a 20-year history of poorly controlled psoriasis and had developed psoriatic arthritis about 3 years prior to admission. He had been treated with methotrexate, azathioprine, etanercept, adalimumab, and golimumab without success due to either poor clinical response or infectious complications largely related to cellulitis from large open skin lesions. For the past 9 months, he had been taking infliximab 3 mg/kg IV infusion every 6 weeks along with erythromycin for prophylaxis against infections with an initial good response for both his skin and joint disease. When his synovitis recurred four months prior to admission, the infliximab dose was increased to 5 mg/kg every 6 weeks. The patient reported initial improvement in his joint and skin symptoms with this dose adjustment. However, over the several weeks prior to admission, he noted increasing muscle pain, stiffness, swelling in his legs, and weight gain.

He denied fevers, weakness, or recent acute respiratory or gastrointestinal illness but did note some night sweats. Past medical history was notable for depression and COPD. Review of systems was otherwise negative. Family history was notable for a poorly characterized autoimmune disease in a grandmother.

Medications on admission were citalopram 40 mg daily, infliximab 5 mg/kg every 6 weeks, naproxen 220 mg BID, hydrocodone/acetaminophen 7.5 mg/325 mg every 6 hours as needed, tramadol 50 mg every 6 hours as needed, folic acid 1 mg daily, fluocinonide 0.05% cream topically for psoriasis, erythromycin 250 mg BID for infection prophylaxis, and topical clotrimazole.

His vital signs at the time of admission were a temperature of 98.2 F, pulse of 87, respiratory rate of 16, blood pressure of 133/73, and oxygen level of 95% on room air. His weight was 63.4 kg which was 7 kg greater than his previous recorded weight 6 months ago. He had mild synovitis of the second and third metacarpophalangeal joints of his left hand. Both wrists were tender to palpation. He also had new leg edema bilaterally with diffuse muscle tenderness to palpation. His legs were warm and erythematous. Muscle strength in upper and lower extremities modestly decreased due to pain in all extremities proximally and distally. Scattered small psoriatic lesions were noted over his bilateral knees, distal legs, and dorsal feet.

Laboratory studies revealed creatinine kinase level of 22 U/L (normal 39–308 U/L) and an aldolase level which was mildly elevated at 11 U/L (normal < 8 U/L). He had a peripheral eosinophilia with eosinophils accounting for up to 30% of his white blood cells. During the hospital stay, his absolute eosinophil count varied between 700 and 2200/*μ*L. He had developed new, mild, normocytic anemia with hemoglobin of 12.0 g/dL and mean corpuscular volume of 94.8 fL. Previous hemoglobin 1 month priorly was 13.8 g/dL. Erythrocyte sedimentation rate was 36 mm/h (normal 0–15) and C-reactive protein was 78.7 (normal 0–10).

Dermatology, rheumatology, and hematology services were consulted. The differential diagnosis included DRESS syndrome (drug rash with eosinophilia and systemic symptoms), malignancy, hypereosinophilic syndrome, inflammatory myopathy, and eosinophilic fasciitis. DRESS syndrome was felt to be unlikely due to the absence of liver involvement. The patient improved with supportive care and outpatient workup was arranged.

**Figure 1:**
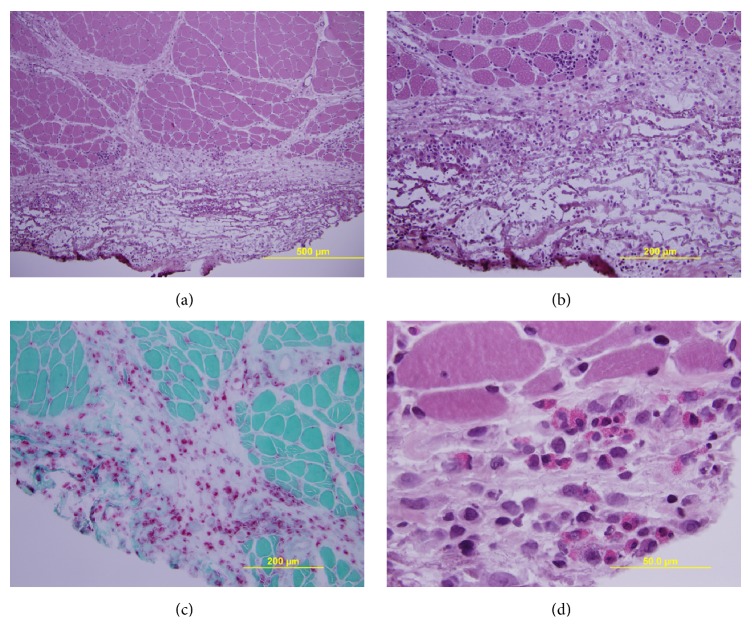
Deltoid muscle biopsy. (a) Low power view showing marked thickening and edema of epimysium and, to lesser extent, perimysium accompanied by diffuse chronic inflammation (H&E stain). There is only minor focal involvement of underlying endomysium. Muscle fascicles are relatively spared. (b) Higher power view of epimysium demonstrating noncohesive chronic inflammation and rarefaction/necrosis of connective tissue (H&E stain). A single necrotic fiber undergoing phagocytosis is observed in the adjacent fascicle. (c) Infiltrates are composed primarily of histiocytes, highlighted as red-staining cells in this preparation (acid phosphatase stain). (d) High power view of more focal collection of eosinophils within the epimysium (H&E stain).

The patient underwent a bone marrow biopsy to evaluate hypereosinophilic syndrome. The biopsy showed hypercellular marrow with marked eosinophilia, but no other significant abnormal cell populations were noted. A scheduled infliximab infusion was performed 2 weeks after hospitalization but had to be stopped due to the development of urticaria during the infusion. A CT scan of the chest, abdomen, and pelvis revealed no evidence of malignancy. The patient underwent an EMG of his right upper extremity to evaluate inflammatory myopathy which showed no definite evidence of a myopathic process. Because of the elevated aldolase level which subsequently increased to 14.3 U/L 3 weeks later, a muscle biopsy of the left deltoid muscle was performed. The biopsy showed diffuse thickening and edema of the epimysium and, to a lesser extent, perimysium, accompanied by a chronic, polymorphic inflammatory infiltrate and foci of fibrinoid necrosis of the involved fibrous connective tissue (Figures 1(a) and 1(b)). The inflammatory cells included histiocytes, lymphocytes, focally prevalent eosinophils, and scattered plasma cells (Figures 1(c) and 1(d)).

These aggregates only rarely infiltrated into the adjacent endomysium. The muscle fibers themselves were relatively spared, with only rare necrotic fibers in perifascicular areas abutting the interstitial inflammation (Figure 1(b)). The findings were indicative of eosinophilic fasciitis. Infliximab was then discontinued and he was started on 60 mg prednisone daily with excellent resolution of his symptoms. Within 24 hours of initiation of prednisone, his muscle pains significantly diminished. Three weeks after initiation of steroid treatment, his edema had resolved, and his eosinophilia normalized.

## 3. Discussion

**Figure 2:**
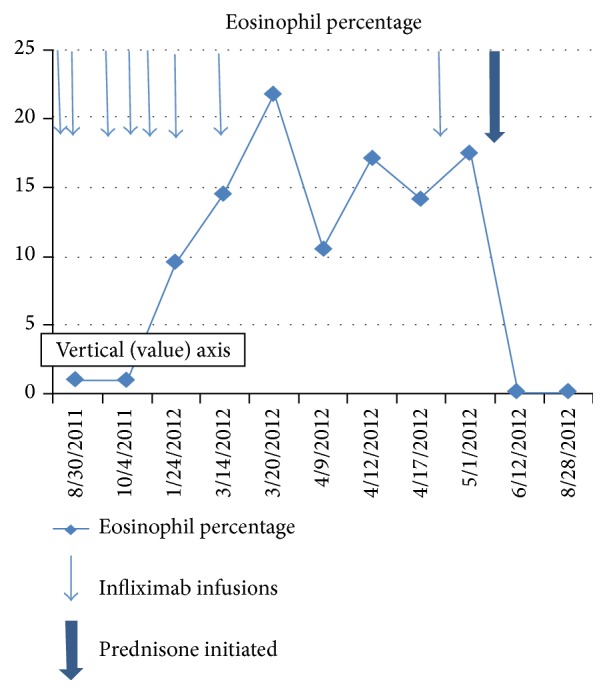
Peripheral eosinophil counts. This graph shows the eosinophil percentage as a function of time. The thin arrows denote the dates of infliximab infusions. The thick arrow shows the initiation of steroid treatment.

We describe a patient who developed biopsy-confirmed eosinophilic fasciitis (EF) on infliximab. Peripheral eosinophilia and typical skin changes occurred after his infliximab dose was increased from 3 mg/kg dose to 5 mg/kg and preceded the development of EF (Figure 2). The pathogenesis of EF is not fully understood, although autoimmune and allergic pathways are thought to be involved. Vigorous exercise,* Borrelia* infection, and malignancy have been associated with EF, although our patient did not have any of these conditions.

The diagnosis of EF is established by clinical, laboratory, and histological findings [1]. Universally accepted international diagnostic criteria are lacking. Clinical manifestations of EF include painful swelling with progressive circumferential induration and thickening of the subcutaneous fat, dermis, and fascia, a finding typically seen in the extremities. All of these features were noted in our patient. The trunk area can also be affected, while the face, fingers, and feet are usually spared. Peripheral eosinophilia may occur in up to 60% of patients. One can also see elevated gamma globulin levels in some cases. Histologically, a full-thickness wedge tissue biopsy of the clinically affected area will show inflammation of the lower subcutis and deep fascia, occasionally extending into the underlying muscle. Muscle biopsy findings in our patient showed inflammation within the perimysial septa and overlying epimysial connective tissue, as is typical of this condition. In a study performed by Wasserman et al., increased eosinophilic chemotactic activity was noted in patients with EF suggesting that elevated peripheral eosinophil activity may have a direct role in this disease process [2].

It seems counterintuitive that a TNF-*α* inhibitor such as infliximab would induce EF. TNF-*α* is a potent chemotactic agent for eosinophils [3] and downregulates eosinophil apoptosis [4]. Several previous cases of eosinophilia have been noted with TNF-*α* inhibitors. Adalimumab was linked with a case of eosinophilic myocarditis [5]. Infliximab has been implicated in a case of eosinophilic cellulitis known as Wells' Syndrome [6], a syndrome of recurrent granulomatous dermatitis with eosinophilia. Infliximab and other TNF-*α* inhibitors can induce autoimmune syndromes such as systemic lupus erythematous [7]. While the mechanisms of these effects are unclear, off-target effects of these drugs or repercussions of antibody formation against these foreign proteins may explain this association.

In regard to treatment, corticosteroids remain the standard therapy for EF, although some patients may improve spontaneously with time. In the setting of malignancy, treating the underlying malignancy is the key to treatment of EF. Other immunologic steroid-sparing medications such as methotrexate, azathioprine, cyclosporine, and rituximab have been tried successfully in the treatment of EF [8]. Interestingly, Khanna et al. reported 3 patients with steroid-resistant EF who improved with addition of infliximab at 3 mg/kg every 8 weeks [9].

In conclusion, we report a case of a 59-year-old male who developed peripheral eosinophilia and subsequent eosinophilic fasciitis during treatment with infliximab for psoriatic arthritis. To our knowledge, this is the first reported case of eosinophilic fasciitis developing from tumor necrosis factor-*α* inhibitors. The mechanism of the development of eosinophilic fasciitis from these medications is unknown, but the description of other eosinophilic syndromes with TNF-*α* inhibitors suggests a possible etiologic link that warrants further study.
